# Investigating Chronic Toxicity, Diet, Patient-Reported Outcomes and the Microbiome in Immunotherapy-Treated Metastatic Melanoma Survivors: A New Frontier

**DOI:** 10.3390/nu18010040

**Published:** 2025-12-22

**Authors:** Margaux Robert, Satabdi Saha, Nazli Dizman, Michelle Rohlfs, Elizabeth Sirmans, Julie Simon, Rodabe N. Amaria, Isabella C. Glitza Oliva, Hussein A. Tawbi, Michael A. Davies, Alexandra Ikeguchi, Karen Basen-Engquist, Keri Schadler, Michael E. Roth, Wenye Song, Xiaotao Zhang, Nadim J. Ajami, Lorenzo Cohen, Jennifer A. Wargo, Christine B. Peterson, Jennifer L. McQuade, Carrie R. Daniel

**Affiliations:** 1 Department of Epidemiology, The University of Texas MD Anderson Cancer Center, Houston, TX 77030, USA; mjrobert@mdanderson.org (M.R.); xiaotao.zhang@mountsinai.org (X.Z.); 2 Department of Biostatistics, The University of Texas MD Anderson Cancer Center, Houston, TX 77030, USA; ssaha1@mdanderson.org (S.S.); cbpeterson@mdanderson.org (C.B.P.); 3 Division of Cancer Medicine, The University of Texas MD Anderson Cancer Center, Houston, TX 77030, USA; ndizman@mdanderson.org; 4 Department of Melanoma Medical Oncology, The University of Texas MD Anderson Cancer Center, Houston, TX 77030, USA; mrohlfs@mdanderson.org (M.R.); esirmans@mdanderson.org (E.S.); rnamaria@mdanderson.org (R.N.A.); icglitza@mdanderson.org (I.C.G.O.); htawbi@mdanderson.org (H.A.T.); mdavies@mdanderson.org (M.A.D.); apikeguchi@mdanderson.org (A.I.); dr.jmcquade@gmail.com (J.L.M.); 5 Department of Surgical Oncology, The University of Texas MD Anderson Cancer Center, Houston, TX 77030, USA; jmgardne@mdanderson.org (J.S.); jwargo@mdanderson.org (J.A.W.); 6 Department of Health Disparities Research, The University of Texas MD Anderson Cancer Center, Houston, TX 77030, USA; kbasenen@mdanderson.org; 7 Division of Pediatrics, The University of Texas MD Anderson Cancer Center, Houston, TX 77030, USA; klschadl@mdanderson.org (K.S.); mroth1@mdanderson.org (M.E.R.); 8 Department of Head & Neck Surgery, The University of Texas MD Anderson Cancer Center, Houston, TX 77030, USA; wsong1@mdanderson.org; 9 Division of Liver Diseases, Icahn School of Medicine at Mount Sinai, New York, NY 10029, USA; 10 Department of Genomic Medicine, The University of Texas MD Anderson Cancer Center, Houston, TX 77030, USA; najami@mdanderson.org; 11 Department of Palliative, Rehabilitation, and Integrative Medicine, The University of Texas MD Anderson Cancer Center, Houston, TX 77030, USA; lcohen@mdanderson.org

**Keywords:** melanoma survivors, immunotherapy, patient-reported outcomes, diet, microbiome

## Abstract

**Background/Objectives**: Immune checkpoint blockade (ICB) therapies have significantly improved outcomes in metastatic melanoma. However, immune-related adverse events (irAEs) and persistent chronic toxicities (CTs) among this emerging survivor population likely influence different facets of quality of life. This study characterized CT, patient-reported outcomes (PROs), diet, physical activity and gut microbiome features in a cohort of long-term survivors with a history of ICB-treated metastatic melanoma. **Methods**: Forty-eight patients with a history of metastatic melanoma who initiated ICB treatment at least 3 years earlier and were not currently on treatment were prospectively enrolled from a melanoma survivorship clinic. Participants completed screening questionnaires for depression, anxiety, diet and physical activity. The gut microbiome was characterized via metagenomic sequencing in a subsample (*n* = 39). Patients’ clinicopathological characteristics and experience of irAEs (during treatment) and CT (persisting >6 months after completion of therapy) were extracted retrospectively from the medical record. **Results**: In the overall cohort, 60% were experiencing CT, while 16% and 20% reported clinically relevant levels of depression and anxiety symptoms, respectively. We observed significant differences in overall gut microbiome composition between survivors with and without CT (*p* = 0.02). Consumption of fruit and vegetables was inversely associated with anxiety (ρ = 0.3, *p* = 0.038). Added sugar consumption was correlated with the severity of experienced symptoms (ρ = 0.4, *p* = 0.003), with pronounced associations across the spectrum of symptoms, including pain, fatigue and shortness of breath (*p* < 0.05). **Conclusions**: These results suggest that CT is experienced by a substantial proportion of ICB-treated metastatic melanoma survivors. Patients experiencing CT also showed distinct microbiome features. However, additional research in prospective settings is needed to confirm these hypotheses.

## 1. Introduction

The advent and approval of immune checkpoint blockade (ICB; e.g., anti-programmed death 1 [PD-1], anti-cytotoxic T-lymphocyte-associated antigen 4 [CTLA-4] and anti-Lymphocyte Activation Gene 3 [LAG-3]) agents has revolutionized the treatment landscape of melanoma, bringing forth an era in which ten-year disease-specific survival in the metastatic setting surpasses 50%, compared with less than 10% previously [1]. Further, analysis of ICB outcomes consistently shows a plateau in survival curves, suggesting long-term benefit and the potential for cures in a substantial proportion of patients [1,2]. Notably, however, improved clinical benefit with ICBs comes with the drawback of immune-related adverse events (irAEs) resulting from immune activation against normal host tissues. In the pivotal phase III CheckMate-067 trial of combination nivolumab and ipilimumab for metastatic melanoma, 96% of the patient population experienced irAEs of any grade, and 59% had grade 3 or higher irAEs [3]. Although many irAEs can be successfully reversed with steroids or other immunosuppressives, real-world data indicate that almost half of irAEs persist for at least 12 weeks, with only 14% of those resolving with long-term follow-up [4]. Lasting effects of persistent irAEs on patients’ quality of life (QoL) are inevitable, and a limited number of studies showed impairments in key QoL domains, such as physical, emotional, cognitive and social functioning, in melanoma survivors after ICB treatment [5,6]. However, it remains unclear whether patient-reported outcomes (PROs), such as anxiety or depression, differ based on the presence of persistent irAEs in survivors. Notably, patient symptoms may influence physical activity and diet, but the nature of those associations remains largely unexplored. Further, we and others have provided strong evidence that the gut microbiome, diet and other host factors are linked to clinical outcomes and irAEs in advanced melanoma [7,8,9,10,11,12,13]. This brings the diet–microbiome axis to the forefront as a potential mediator of the link between PROs and persistent toxicities, defining a novel aspect of investigation that has not been explored in a survivor population.

As ICBs have now been approved in numerous disease types, the long-term ICB survivor population is rapidly emerging and expanding, raising new questions about their life after treatment. Given that diet and lifestyle influence QoL in cancer survivors [7,11,14,15], and that the gut microbiome is associated with both response and irAEs in ICB [8,9,12,13], we employed a multi-disciplinary and integrated approach to characterize the complex interplay between PROs, diet, physical activity and the gut microbiome in a cohort of metastatic melanoma survivors with and without persistent chronic toxicities (CTs) from prior ICB therapy.

## 2. Materials and Methods

### 2.1. Study Population and Design

This cross-sectional study of patients with a previous history of treated metastatic melanoma was conducted as part of a prospective study of lifestyle factors, PROs and the gut microbiome in melanoma patients at The University of Texas MD Anderson Cancer Center Melanoma and Skin Center in Houston, Texas [7]. For this sub-cohort of 48 stage IV melanoma survivors, 47 patients were treated with ICB, and 1 patient achieved durable complete response with chemotherapy followed by targeted therapy. Survivors were further defined as having previously been treated with systemic therapy, with treatment initiation at least 3 years prior, and were no longer on any active cancer treatment.

Patients provided voluntary and informed consent to all research procedures, including biospecimen collection, under Institutional Review Board (IRB)-approved protocols between November 2021 and May 2022. Survey modules assessing PROs, diet and physical activity were administered at a single time point when patients presented for their survivorship follow-up, primarily via secure web-based format or by paper copy, according to the patient’s preference. A single fecal sample to characterize the gut microbiome was collected concurrently. Sociodemographic and disease characteristics were extracted from their electronic medical record, including age, sex, race, body mass index (BMI) at time of survey, melanoma stage, the number of lines of therapy and time since the last treatment. Body mass index (BMI) was classified into 3 categories as normal, overweight and obese according to the WHO reference values.

### 2.2. Assessment of irAEs and Chronic Toxicity

irAEs were assessed retrospectively using data extracted from electronic health records. Patients who developed at least one irAE during or after immunotherapy treatment were identified. Each irAE was then categorized and graded using the Common Terminology Criteria for Adverse Events (CTCAE) v5.0 [16]. The highest CTCAE grade experienced by the patient was recorded. irAE treatment (i.e., steroids, biologics, hormonal replacement) was extracted from the chart. Chronic toxicity (CT) was defined as an irAE leading to persistent symptoms or requiring ongoing follow-up or therapy for at least 6 months after therapy completion. Patients with persistent CT were further categorized into 3 categories based on organ site involvement: any CT (including hormonal only, non-hormonal only and both), hormonal CT (hormonal only) and other CT (including non-hormonal CT and both hormonal and non-hormonal).

### 2.3. Fecal Microbiome Sequencing and Analysis

Stool samples were collected by patients at home using an OMNIgene GUT (OMR-200, DNA Genotek, Ottawa, ON, Canada) mailable kit with detailed instructions provided in the clinic. Patients returned stool samples via pre-stamped, prelabeled return envelope to the receiving lab at MD Anderson or brought them to the clinic. Upon receipt, samples were assessed, labeled and logged within the database, then transferred to −80 °C storage. Genomic DNA was extracted from stool samples using the QIAmp Fast DNA Stool Mini Kit (Qiagen, Venlo, The Netherlands) with an additional bead-beating lysis step. Briefly, each sample tube contained the stool sample, one 3.2-mm steel bead, approximately 150 mg of zirconium beads, and lysis buffer. Samples were bead-beaten for a total of 8 min at 3800 rpm (BioSpec, Bartlesville, OK, USA) to ensure efficient bacterial lysis, after which DNA extraction proceeded according to the manufacturer’s protocol. Extracted genomic DNA was prepared into individual sequencing libraries using the Illumina DNA Prep Kit (Illumina, San Diego, CA, USA). Libraries were pooled and sequenced on the Illumina NovaSeq 6000 platform using the NovaSeq 6000 v1.5 Reagent Kit. Sequencing generated approximately 20 million reads per sample. Paired-end sequencing was performed using a 2 × 150 bp read protocol in accordance with the manufacturer’s instructions. Gut microbiome profiles were generated using Metagenomic Phylogenetic Analysis (MetaPhlAn) 4 [17], a publicly available computational tool.

### 2.4. Assessment of Patient-Reported Outcomes

Depressive symptomatology was assessed with the Center for Epidemiological Studies-Depression (CES-D) Scale [18], a 20-item self-administered questionnaire. The total possible score ranges from 0 to 60, with a higher score indicating higher depressive symptomatology. Participants were also classified according to the presence of depressive symptoms using the commonly employed cutoff of 16 [18]. State anxiety was assessed with the state subscale of the State-Trait Anxiety Inventory (STAI-S) Form Y [19], comprising 20 items. The total score ranges from 20 to 80, a higher score reflecting a greater tendency to anxiety. Participants were classified according to the presence of anxiety symptoms using the commonly used cutoff of 40 [19]. The MD Anderson Symptom Inventory (MDASI) [20] was used to evaluate symptoms, their severity and the interference with daily living caused by those symptoms. The MDASI comprises 19 items, 13 related to symptom severity and 6 related to interference. For both subscales, scores range from 0 to 10, with higher scores indicating greater severity of symptoms and greater interference with living, respectively. Additionally, the 13 symptoms were classified as mild (score ≤ 4), moderate (score of 5 or 6) and severe (score ≥ 7) [20]. For each scale, patients with more than 2 missing items (CES-D, *n* = 5; STAI-S, *n* = 4; MDASI, *n* = 3) were excluded from analyses. For patients with 2 or fewer missing items, the missing items were imputed by item-mean before calculating the total score [21].

The CES-D scale showed acceptable internal consistency (Cronbach’s α = 0.77), and the STAI and the 2 MDASI subscales (severity of symptoms and interference with daily living) showed excellent internal consistency (Cronbach’s α = 0.93, 0.94 and 0.98, respectively).

### 2.5. Assessment of Diet and Physical Activity

Participants also completed screening questionnaires for diet and physical activity, including the National Cancer Institute Dietary Screener Questionnaire (NCI-DSQ), as previously described [7], and the Godin Leisure-Time Exercise-Questionnaire [22]. The NCI-DSQ includes 26 food item queries used to derive estimated consumption of the following food groups and nutrients: calcium, fiber, dairy, fruits, vegetables and legumes (excluding French fries), whole grains, total added sugars, and added sugars from beverages [23]. A single qualitative measure of the frequency of red and processed meat consumption is not included in the validated scoring algorithm and was thus not part of our analysis [23]. Frequency responses were manually reviewed to identify improbable or implausible patterns and responses. Patients who did not respond to more than 2 food item queries (*n* = 6) on the DSQ were excluded from the analyses. Missing data were imputed by mode for patients with 1 (*n* = 13) or 2 (*n* = 3) missing responses, as previously described [7]. Statistical outliers were verified using the 1.5 IQR rule. The chi^2^ test showed no difference in the proportion of outliers between participants experiencing and not experiencing CT (*p* = 0.269). Similar results were found for the different CT types (*p* = 0.385). Three levels of physical activity were defined: insufficiently active (score < 14 units), moderately active (14 ≤ score < 24 units) and active (score ≥ 24 units) [24].

### 2.6. Statistical Analyses

The analytic cohort for this study included 48 patients. After single mean-item imputation, 43 participants contributed data for the CES-D, 44 for the S-STAI, 45 for the MDASI, 40 for the Godin and 42 for the NCI-DSQ. Thirty-nine patients contributed to the gut microbiome analysis.

Sociodemographic characteristics, lifestyle assessment, clinical factors and PROs were summarized as mean ± standard deviation for continuous variables and number (frequency) for categorical variables. Variables were then compared according to CT status using the Mann–Whitney U test for continuous variables and $\chi^{2}$ or Fisher’s exact test for categorical variables, using the Kruskal–Wallis test followed by the Dwass–Steel–Critchlow–Fligner test for multiple comparison. Relationships between consumption of derived food groups and PROs were assessed with Spearman correlations. A priori hypotheses centered on potential differences related to CT led us to further examine the pattern of relationships between variables in each subgroup (survivors with and without CT). Similar analyses were conducted distinguishing between participants with only CT and participants with non-hormonal CT. *p*-values were adjusted for multiple comparisons using the Benjamini–Hochberg (BH) method, and we report both the original *p*-values and BH-adjusted q-values, as appropriate.

To characterize the diversity of bacteria in fecal samples (alpha diversity), we calculated the Inverse Simpson index on rarefied count data using the R package phyloseq (version 1.50.0) [25]. The Wilcoxon rank-sum test was used to compare alpha diversity between groups based on CT or hormonal CT status. For continuous PRO scores, we performed linear regression analysis to assess the association between the alpha diversity index and the individual PRO scores. To facilitate interpretation, we also visualized the relationships using LOESS plots in R. We used stacked bar plots to visualize the microbiome composition of stool samples and to visually represent diversity across samples. We computed per-sample phylum-level count proportions and then used the R package corncob to fit a beta-binomial regression model [26], testing the association between each phylum’s proportional abundance and CT group. For beta diversity analysis, we generated PCoA plots using Weighted Unifrac distances [27]. To assess global differences in microbiome composition between groups, we performed permutational analysis of variance (PERMANOVA) using the adonis2 function in the R package vegan [28]. To identify specific taxa that were differentially abundant between patient groups, differential abundance analysis using the R package MaAsLin2 [29] was computed. MaAsLin2 uses generalized linear models to identify differentially abundant taxa and can handle sparse count data, and models were adjusted for age, sex and BMI. The alpha and beta diversity analyses along with the differential abundance analysis using MaAsLin2 were performed on rarefied count data. We applied prevalence filtering, retaining only taxa with non-zero abundance in at least 5% of samples, and excluding rarer taxa from downstream analysis. The BH method to adjust the raw *p*-values [30] was applied for multiplicity correction. The FDR cutoff was set at 0.05. In addition, to filter out spurious associations, the species were filtered to have a log fold change (LFC) > 1 and a prevalence of ≥25% [31]. All tests of statistical significance were 2-sided, and nominal significance was set at 5%. Statistical analyses were performed using R and SAS version 9.4 software (SAS Institute, Inc., Cary, NC, USA).

## 3. Results

### 3.1. Patients’ Characteristics

We herein describe a unique population, shown in Table 1, of 48 long-term metastatic melanoma survivors, 47 of whom were treated with ICB and followed for 4.3 (range 1.1–8.5) years from last treatment initiation and 3.2 (range 0.8–6.8) years from last treatment completion. The majority of participants were male (75%), and the average number of lines of treatment was 2.1 ± 1.8. Approximately 80% of the survivors experienced irAEs and 60% CT, comprising 47% hormonal-only CT, and 16.7% other CT (non-hormonal or both hormonal and at least one non-hormonal). Among ICB-treated patients with CT (*n* = 28), 39.5% had two or more CTs. The majority of patients experiencing CT had been treated with combination ICBs (anti-PD-1 and anti-CTL-4 antibodies) (Supplemental Table S1). The most common irAEs were endocrinopathies (75%), followed by diarrhea/colitis (40%) (Supplemental Figure S1). Among patients experiencing CT, the most common CTs were hypothyroidism (72.4%) and adrenal insufficiency (34.5%) (Supplemental Figure S1). Among patients with CT, 17.2% required ongoing treatment with systemic steroids (not including hydrocortisone), and 79.3% required ongoing treatment with hormonal replacement therapy.

Within the clinical screening range, 16.3% of the participants reported depressive symptoms, 20.5% reported anxiety symptoms and 11.9% reported both concurrently. MDASI scores indicated overall mild symptoms (1.6 ± 1.7) and mild interference of symptoms with daily life (2.4 ± 2.1) [20]. More than 80% of patients rated each of the 13 symptoms as mild. Notably, a few participants reported moderate fatigue (12.5%), shortness of breath (6.7%), difficulty remembering (8.9%), drowsiness (6.7%) and numbness/tingling (8.9%) (Supplemental Table S2). Around 12.5% of the cohort reported being physically inactive, and 39.6% were classified as obese. The reported frequency of consumption and estimated intake of fruits, vegetables and legumes, and whole grains were generally below the recommended levels for chronic disease prevention, while consumption of added sugars was above recommended levels [23,32]. Mean dietary fiber consumption was estimated to be around 18 g per day, similar to our prior report among melanoma patients in the active treatment setting [7] and also below recommended levels for the general adult population [32]. Around 20% of survivors reported probiotic supplement use, which was lower than what we previously observed (~30%) among patients in the active treatment setting [7].

### 3.2. Patients’ Characteristics by Chronic Toxicity Prevalence

**Figure 1:**
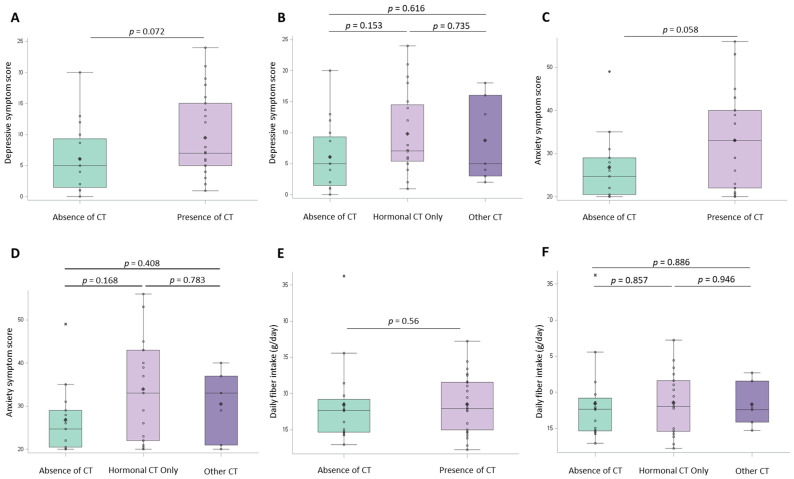
Patient-reported outcome measures of anxiety, depression and dietary fiber intake by prevalence of chronic toxicity among metastatic melanoma survivors. (**A**–**F**), box plot comparing patient-reported outcomes (PRO) and diet by prevalence of chronic toxicities (CTs). Median, mean (diamond-shaped), interquartile range (box) and whiskers representing data variability are shown. Box plot comparing the following: (**A**), Depressive symptom score by prevalence of CT; groups include absence of CT (*n* = 16) and any CT (*n* = 27) (*p* = 0.072, by Mann–Whitney U test). (**B**), Depressive symptom score by prevalence of CT. Groups include no CT (*n* = 16), hormonal CT only (*n* = 20) and other CTs (*n* = 7). Statistical comparisons include no CT versus hormonal CT only (*p* = 0.153), no CT versus other CT (*p* = 0.616) and hormonal CT only versus other CT (*p* = 0.735), analyzed using the Kruskal–Wallis test followed by the Dwass–Steel–Critchlow–Fligner (DSCF) test for pairwise comparisons. (**C**), Anxiety symptom score by prevalence of CT; groups include absence of CT (*n* = 17) and any CT (*n* = 27) (*p* = 0.058, by Mann–Whitney U test). (**D**), Anxiety symptom score by prevalence of CT. Groups include no CT (*n* = 17), hormonal CT only (*n* = 20) and other CT (*n* = 7). Statistical comparisons include no CT versus hormonal CT only (*p* = 0.168), no CT versus other CT (*p* = 0.408) and hormonal CT only versus other CT (*p* = 0.783), analyzed using the Kruskal–Wallis test followed by the DSCF test for pairwise comparisons. (**E**), Daily dietary fiber intake (in g) by prevalence of CT; groups include absence of CT (*n* = 16) and any CT (*n* = 26) (*p* = 0.056, by Mann–Whitney U test). (**F**), Daily dietary fiber intake (in g) by prevalence of CT. Groups include no CT (*n* = 16), hormonal CT only (*n* = 19) and other CT (*n* = 7). Statistical comparisons include no CT versus hormonal CT only (*p* = 0.857), no CT versus other CT (*p* = 0.886) and hormonal CT only versus other CT (*p* = 0.946), analyzed using the Kruskal–Wallis test followed by the DSCF test for pairwise comparison.

Given that toxicities could have an impact on daily life and mental health, we explored the association between CT and PROs, diet and physical activity. There were no demographic differences between participants experiencing and not experiencing CT (Table 2). Results were similar across most PROs, diet components, and physical activity between those who did and did not experience CT. In particular, there was no difference based on CT in consumption of dietary fiber (*p* = 0.56) (Figure 1A,B) or in symptom experience and impact on daily living (*p* = 0.815 and *p* = 0.626, respectively). When examining cut points within the clinical screening range of anxiety or depression based on CT status, the proportion of patients meeting the depression criteria (22.2% vs. 5.8%) or anxiety criteria (29.6% vs. 6.5%) was higher among survivors with versus without CT, respectively. Survivors with CT reported numerically higher depression and anxiety scores than those without CT, although these differences did not reach statistical significance (*p* = 0.072 and *p* = 0.058, respectively; Figure 1C–F). No clear differences were noted based on CT type, i.e., hormonal versus non-hormonal.

### 3.3. Associations Between Patient-Reported Outcomes, Diet and Physical Activity

**Figure 2:**
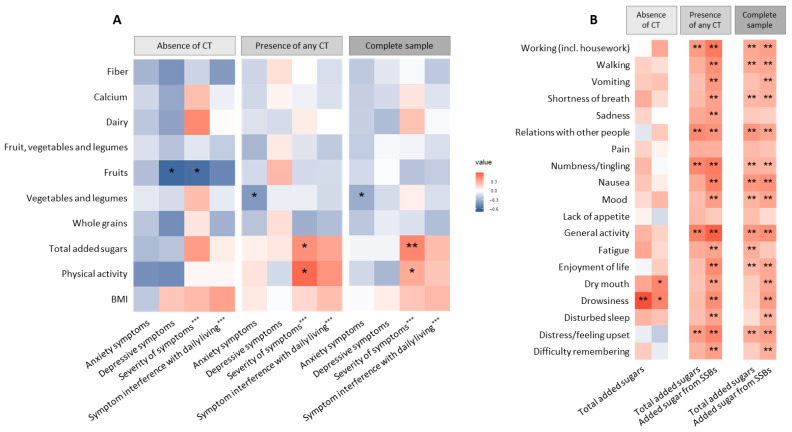
Associations between patient-reported outcomes, diet and physical activity by prevalence of chronic toxicity among metastatic melanoma survivors. Abbreviation: CT, chronic toxicity. (**A**), Spearman correlation of patient-reported diet (derived food groups) with patient-reported outcome measures of symptoms, anxiety, depression and physical activity in patients without CT (*n* = 19), with any CT (*n* = 29) and in the complete sample (N = 48). (**B**), Spearman correlation of MD Anderson Symptoms Inventory (MDASI) items with total added sugars and added sugars from beverages in patients without CT (*n* = 19), with any CT (*n* = 29) and in the complete sample (N = 48). * Nominal *p*-value < 0.05, ** nominal *p*-value < 0.05 and q-value < 0.2 by false discovery rate correction for multiple testing (FDR), *** as measured by the MD Anderson Symptom Inventory.

**Figure 3:**
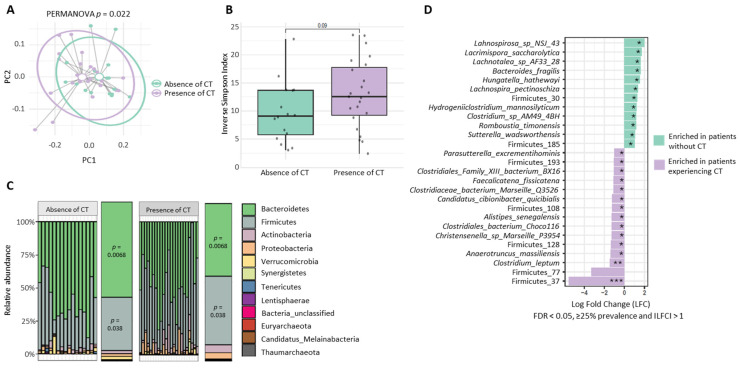
Microbiome characteristics by prevalence of chronic toxicity among metastatic melanoma survivors (*n* = 39). (**A**), Principal coordinates analysis (PCoA) plot based on UniFrac distances for beta diversity showing differences in microbial community composition between patients with and without chronic toxicities. Each point represents a sample, and colors indicate group membership. Separation between clusters reflects differences in community structure. A PERMANOVA test was conducted to assess group-level differences, yielding a significant result (PERMANOVA *p* = 0.022 *). (**B**), Box plot of alpha diversity (Inverse Simpson index) across chronic toxicity groups, reflecting within-sample microbial diversity. Comparisons illustrate differences in diversity between the two groups. A Wilcoxon rank-sum test was used to compare alpha diversity between the two groups, yielding a *p*-value of 0.09. (**C**), Stacked bar plot of phylum-level microbial composition across conditions. For each condition, two bars are shown side by side: the left bar represents the mean relative abundance calculated per sample, and the right bar represents the overall group-level average. Colors correspond to different phyla. *p*-value for the overall Firmicutes/Bacteroidetes ratio = 0.011). (**D**), Horizontal Forest plot of MaAsLin2-derived effect sizes for microbial features significantly associated with chronic toxicity (CT) status. Each point shows the estimated coefficient (log fold change) for features meeting the criteria of |log FC| > 1, FDR < 0.05 and prevalence ≥ 25%. Points are colored by direction of association: purple for features enriched in CT-positive (“Yes”) samples and green for those enriched in CT-negative (“No”) samples. * *p*-value < 0.001, q-value < 0.001; ** *p*-value = 0.015, q-value = 0.021; *** *p*-value < 0.001, q-value = 0.0011.

We hypothesized that symptom burden, including depression and anxiety, may influence the patient’s ability to engage in healthy eating and other lifestyle behaviors, and that these associations could differ by CT status. We observed a number of significant correlations between symptom scores, diet and physical activity (Figure 2). Overall, we found that consumption of vegetables and legumes (excluding French fries) was inversely correlated with anxiety (Spearman ρ = −0.32, *p*-value = 0.038, q-value = 0.514), and symptom severity was positively correlated with total added sugars (ρ = 0.45, *p* = 0.003, q = 0.121). In addition, a positive correlation between symptom severity and physical activity levels was nominally significant (ρ = 0.32, *p* = 0.046, q = 0.514) (Figure 2A, Supplemental Table S3). The impact of symptoms on daily living was positively correlated with consumption of added sugars from beverages (ρ = 0.40, *p* = 0.009, q = 0.121). To further explore this, we assessed the associations between added sugars and the different items of the MDASI (Figure 2B, Supplemental Table S4). Total added sugar was positively correlated with the severity of fatigue, distress, shortness of breath, mood, nausea, numbness and the interference of symptoms with enjoyment of life, relations with others, general activity and walking. Similarly, added sugar intake from beverages was positively associated with multiple symptoms and their impact on daily living (Figure 3B). Stratified analyses showed that these associations may be largely driven by the participants experiencing CT (Figure 2A,B).

### 3.4. Overall Gut Microbiome Composition and Phylum-Level Associations with CT

Given that dietary fiber intake and healthy dietary patterns shape the gut microbiome and have been associated with improved ICB response [7,11] and potentially lower rates of irAEs [11] in melanoma patients, we sought to assess the composition of the microbiome in metastatic melanoma survivors living with and without CT (Figure 3). We conducted analyses of both alpha and beta diversity (Figure 3A,B) in a subgroup of 39 metastatic survivors with available stool for sequencing. The inverse Simpson index possibly trended higher in those with CT (*p* = 0.09, Figure 3B), while beta diversity analysis based on UniFrac distances suggested a significant difference in gut microbiome composition between subjects with and without CT (*p* = 0.022) (Figure 3A). To confirm whether the differences in beta diversity were driven by differences in abundance we repeated the analysis using Jaccard distance, which only accounts for the presence or absence of a feature, and found consistent results. The stacked bar plot of average phylum compositions shown in Figure 3C revealed clear differences in the relative proportions of Firmicutes and Bacteroidetes between the CT groups. We therefore fitted a beta-binomial regression model to formally test these observations. Participants experiencing CT had approximately 50% lower odds of greater relative abundance of Bacteroidetes compared to those without CT (odds ratio [OR] = 0.50; *p* = 0.0068) and 61% higher odds of greater relative abundance of Firmicutes (OR = 1.61; *p* = 0.038). Overall, the Firmicutes/Bacteroidetes ratio was about twice as high in those experiencing CT (OR of ratio = 1.97, *p* = 0.011).

### 3.5. Differential Species Abundance Across CT Groups

We next focused on differential abundance analysis to identify specific microbial features that differed between survivors with and without CT (Figure 3D). Interestingly, in survivors experiencing CT, we observed strong positive enrichment for two unclassified Firmicutes species and *Clostridium leptum*, which has previously been associated with ICB response [13,33,34], along with other members of the Clostridiaceae family [13]. *Anaerotruncus massiliensis,* a member of the *Oscillospiraceae* family involved in mucin degradation, which has been linked to ICB response and irAEs [35], was also enriched among survivors experiencing CT (Figure 3D). Notably, in the group of survivors without CT, four species from the Lachnospiraceae family were differentially abundant, namely *Lachnospira_sp_NSJ_43*, *Lacrimispora saccharolytica*, *Lachnotalea sp AF33 28* and *Lachnospira pectinoschiza* (Figure 3D). Beta-binomial regression was performed to further verify whether the overall relative abundance of Lachnospiraceae [12] differed between survivors experiencing and not experiencing CT, but no statistically significant difference was observed (*p* = 0.539).

### 3.6. Association of Gut Microbiome with PROs

To explore the microbiome’s association with PROs, we carried out alpha and beta diversity analyses as described above using groups defined by PRO status (Supplemental Figure S2). Despite limited sample size in the defined subgroups, beta diversity analyses suggested a trend toward a potential difference for depression scores (*p* = 0.068), but not for anxiety scores (*p* = 0.12), severity of symptoms (*p* = 0.571) or their interference with living (*p* = 0.466), dietary factors (including fiber (*p* = 0.777), total added sugars (*p* = 0.2), added sugars from beverages (*p* = 0.11)) or physical activity scores (*p* = 0.954) (Supplemental Figure S2B). Alpha diversity analysis revealed no significant associations with PROs. Differential abundance analysis identified no notable associations between specific species-level taxa and individual PROs (Supplemental Figure S2A).

## 4. Discussion

This study aimed to characterize PROs, diet, physical activity and gut microbiome variation in a cohort of metastatic melanoma survivors previously treated with ICB. Within this study, 80% of survivors experienced irAEs, and 60% were living with CT, including 44% with hormonal CT. Given that toxicity and tumor response often co-occur, this high rate of toxicity is not unexpected in this cohort of metastatic melanoma survivors and is in line with prior reports [4,36,37]. Despite the high rate of CT, our results showed no differences in symptom severity or interference with living, BMI, physical activity or diet between patients experiencing and not experiencing CT. This may be because the vast majority of CT was hormonal only, which is treated with physiologic replacement of affected hormones (e.g., thyroid replacement for hypothyroidism or hydrocortisone for adrenal insufficiency), and is therefore unlikely to cause any significant symptom burden. Over time, all irAEs that met the CT definition improved to grade 1–2. However, our results suggest a trend toward higher depression and anxiety symptom scores in survivors experiencing CT.

Although in the overall population, MDASI scores indicated mild symptoms and interference of these symptoms with daily living, around 20% of patients demonstrated symptoms of anxiety, and around 16% symptoms of depression. The prevalence of depression in our sample was similar to that reported in a nationally representative sample of cancer survivors, including survivors of colorectal, breast or genitourinary cancers (19.7%), but anxiety was reported at a considerably higher rate than in our sample (45.8%) [38]. However, our findings are in line with a previous meta-analysis reporting similar prevalences of anxiety and depression (20% and 14%, respectively) among stage III and stage IV melanoma survivors [39], which is similar to those of the general US population [40]. Melanoma-related anxiety and depression are suggested to peak at diagnosis and resolve over time, whereas anxiety and depression from other sources remain constant [41]. However, specifically querying disease-related worry is an important future next step in this emerging survivorship group where long-term outcomes are unclear.

Most survivors were physically active, but 40% were classified as obese, a factor that has paradoxically been found to be associated with an improved response to ICB [42]. In addition, consumption of dietary fiber and fiber-rich foods, such as vegetables and legumes, was lower than the recommendations, while intake of added sugars was higher than the recommendation. This was unexpected to us given that we and others have previously demonstrated that higher dietary fiber intake and healthier dietary patterns are associated with higher rates of ICB response in melanoma patients in the active treatment setting [7,11], a trend that did not appear to extend to this survivor cohort of treatment responders. Unfortunately, we did not have an assessment of lifestyle factors for this cohort of patients at the time of treatment initiation. However, our findings are consistent with the prior literature in pan-cancer studies in which a large proportion of cancer survivors report limited changes toward positive health behaviors in general [40,43]. Results from the American Cancer Society’s studies of cancer survivors-II (SCS-II) showed that only 40% of cancer survivors reported positive changes in healthy eating behaviors [40].

Analyses of interactions between PROs, physical activity and diet showed that anxiety symptoms were negatively correlated with vegetable and legume intake, consistent with existing data reporting that anxiety is associated with an unhealthy diet [44], including lower intakes of legumes and cereals and higher intakes of sweets or readymade meals [45]. In addition, we observed that severity of symptoms and their interference with life were positively correlated with total added sugar consumption, particularly among participants experiencing CT. Though both conditions were assessed at the same time in this study, the correlation appears to be in line with the previous literature showing that emotional eating, characterized by increased intake of sweet and high-fat food [46], can be a way to cope with negative aspects of life [47]. Interestingly, higher severity of symptoms, in particular fatigue and pain, correlated with physical activity, consistent with existing recommendations for fatigue management [48].

Collectively, microbiome analyses of beta diversity, phylum-level associations and individual taxa revealed distinctions in community composition between patients experiencing and not experiencing CT. Several independent melanoma cohorts previously demonstrated that gut microbiome features in the Oscillospiraceae and the Lachnospiraceae families are associated with patient’s response to ICB [7,12,13]. As the vast majority of the patients in the current study were ICB responders by definition, it was not surprising that similar favorable ICB response-associated bacteria were also present in our population of survivors, across both groups of participants, with and without CT. Interestingly, several specific taxa within the family Lachnospiraceae, a feature previously linked to both response and irAEs in the treatment setting [12], were lower in survivors with CT versus without CT, hinting that this signal may not be consistent for irAEs and CT. However, a prospective, longitudinal study would be required to further investigate this observation and define an intra-individual shift or temporal trend. Other microbiota enriched among survivors with CT, including enrichment of two unclassified Firmicutes species and members of the Clostridiaceae and Oscillopsiraceae families [7,13,33,34,35], highlight the role of the microbiome in the immune response to ICB, including anti-tumor immune response and irAEs. Although we were unable to assess this in our cross-sectional study of survivors, depression, anxiety and microbiome composition are also likely intertwined with bidirectional associations that may be amenable to diet and lifestyle interventions [49,50].

This study is one of the first to broadly characterize this new cohort of metastatic melanoma survivors, and the multi-dimensional analysis has generated several hypotheses for future studies. However, we acknowledge some limitations. The total sample size for the stool microbiome (*n* = 39) may have limited the variability within and between key subgroups to detect associations. In addition, the cross-sectional design prevents strong or causal conclusions about the direction of the observed associations. The NCI-DSQ, which focuses on 26 pre-specified food and beverage items, is useful to identify individuals in need of intervention, while minimizing patient burden through its brevity. The NCI-DSQ is limited in its ability to capture patients’ overall eating patterns or to quantify nutrient profiles, which could be highly informative in this setting. This is particularly relevant given that several survivors also reported following a diet they defined as “other” when presented with the criteria of omnivore, vegetarian or vegan. Levels of depression and anxiety symptoms were assessed with commonly used and validated measures [18,19]. However, these questionnaires do not substitute for a clinical diagnosis, and we cannot exclude the possibility of false positives or false negatives. Additionally, information on participants’ prior history of depression or anxiety was not available. Further studies should consider exploring these associations in larger, longitudinal studies with more comprehensive assessments. Lastly, patients who chose to participate in this single-center study may not be entirely representative of the broader population of metastatic melanoma survivors.

## 5. Conclusions

Our study is one of a few describing the experience and landscape of survivors of ICB-treated metastatic melanoma. Our study encompasses assessment of CT, PROs, diet and physical activity, and is the first to explore the gut microbiome in this setting. Our results indicate that more than half of the survivors experienced chronic toxicity. Participants experiencing CT exhibited potential differences in the composition and structure of their gut microbiomes, compared to survivors without CT. However, these associations should be further explored in longitudinal studies that incorporate aspects of microbiome function to elucidate the clinical implications of these differences. Although physical symptoms were similar between ICB-treated metastatic melanoma survivors living with and without CT, we observed a trend toward higher overall depression and anxiety symptom scores in survivors with CT, as well as potential differences in the composition of their gut microbiome. Based on these findings, we propose the hypothesis that in metastatic melanoma survivors, CT may lead to poorer mental health, which in turn might affect diet. Further research is needed to determine the directionality and causality of these postulated associations. Additionally, investigations are warranted in survivors of other cancers, as well as earlier stage melanoma, currently being treated with ICB. Together, our results highlight the importance of considering mental health and diet in the follow-up of ICB-treated cancer survivors. This is particularly important as unhealthy behaviors are associated with increased risk of comorbidities and second cancers [51,52,53]. Further prospective studies with larger sample sizes and additional measures of QoL are needed to further explore the strength and direction of these associations.

## Figures and Tables

**Table 1:** Characteristics of metastatic melanoma survivors.

	Mean ± SD or n (%)
Patient and clinical characteristics (N = 48)	
Age, years ^1^	63.6 ± 12.32
Sex	
Female	12 (25.0)
Male	36 (75.0)
Ethnicity	
Hispanic	2 (4.2)
White non-Hispanic	46 (95.8)
Body mass index category ^1^	
Normal	14 (29.2)
Overweight	15 (31.2)
Obese	19 (39.6)
Melanoma stage ^2,3^	
M1a	5 (10.4)
M1b	13 (27.1)
M1c	13 (27.1)
M1d	17 (35.4)
Number of lines of treatment ^2^	2.1 ± 1.7
Immunotherapy treatment, *n* = 47 ^2^	
Combination therapy	24 (50.0)
Monotherapy	22 (45.8)
Other	2 (4.2)
Time since last treatment completion, years ^2^	3.2 ± 1.8
irAEs during treatment ^2^	
No	10 (20.8)
Yes	38 (79.2)
Chronic toxicities persisting after treatment	
No	19 (39.6)
Hormonal toxicities	21 (43.7)
Other toxicities	8 (16.7)
Patient-reported outcome and lifestyle ^1^	
Depressive symptoms ^4^, *n* = 43	
No	36 (83.7)
Yes	7 (16.3)
Anxiety symptoms ^5^, *n* = 44	
No	35 (79.5)
Yes	9 (20.5)
MDASI score, *n* = 45	
Severity of symptoms ^6^	1.6 ± 1.7
Interference with daily living ^7^	2.4 ± 2.1
Physical activity level ^8^, *n* = 40	
Insufficiently active/sedentary	5 (12.5)
Moderately active	2 (5)
Active	33 (82.5)
Daily dietary intake ^9^, *n* = 42	
Dietary fiber, g	18.5 ± 4.6
Calcium, mg	1036.7 ± 165.1
Dairy, cup equivalent	1.6 ± 0.4
Fruit, vegetables and legumes, cup equivalent	3.1 ± 1.1
Fruits	1.0 ± 0.7
Vegetables and legumes	1.9 ± 0.8
Whole grains, ounce equivalent	0.8 ± 0.4
Total added sugars, tsp	17.2 ± 5.9
Added sugars from beverages	6.9 ± 4.8
Diet type, *n* = 45	
Vegetarian	1 (2.2)
Vegan	0 (0.0)
Omnivore	15 (33.3)
Other, undefined	29 (64.5)
Probiotic use, *n* = 44	
No	35 (79.5)
Yes	9 (20.5)

Abbreviations: MDASI, MD Anderson Symptom Inventory; tsp, teaspoon. ^1^ Assessed at study enrollment. ^2^ Assessed retrospectively using data extracted from electronic health records. ^3^ AJCC 8 staging: M1a: metastatic only to skin, soft tissue and/or non-regional lymph nodes; M1b: metastatic to lung ± sites that define M1a; M1c: metastatic to any other distant organ other than central nervous system; M1d: metastatic to central nervous system. ^4^ Center for Epidemiological Studies-Depression (CES-D): a score ≥ 16 was used to define the presence of depressive symptoms. ^5^ State-Trait Anxiety Inventory, state subscale (STAI-S): a score ≥ 40 was used to define the presence of anxiety symptoms. ^6^ Score ranging from 1 to 13, with higher scores indicating greater severity of symptoms. ^7^ Score ranging from 1 to 6, with higher scores indicating greater interference with living. ^8^ Godin–Shephard Leisure-Time Physical Activity Questionnaire: a score < 14 was defined as insufficiently active, between 14 and 24 was defined as moderately active and ≥24 was defined as active. ^9^ Frequency of intake reported over a 30-day period via the National Cancer Institute Dietary Screener Questionnaire (NCI-DSQ).

**Table 2:** Characteristics of metastatic melanoma survivors by prevalence of chronic toxicity (N = 48).

	No CT (*n* = 19)	Any CT (*n* = 29)	Hormonal CT Only (*n* = 21)	Other CT ^1^ (*n* = 8)	*p*-Value No CT vs. Any CT ^2^	*p*-Value No CT vs. Hormonal CT Only ^3^	*p*-Value No CT vs. Other CT ^3^	*p*-Value Hormonal CT Only vs. Other CT ^3^
Body mass index, kg/m ^2^	28.11 ± 4.88 ^4^	28.07 ± 5.19	27.21 ± 5.39	30.34 ± 4.09	0.966	0.761	0.284	0.284
Depressive symptom score ^5^	6.04 ± 5.5	9.48 ± 6.5	9.75 ± 6.57	8.71 ± 6.73	0.072	0.153	0.616	0.735
Anxiety symptom score ^6^	26.7 ± 7.63	33.02 ± 11.12	33.92 ± 12.15	30.43 ± 7.61	0.058	0.168	0.408	0.783
MDASI score								
Severity of symptoms ^7^	1.33 ± 1.02	1.73 ± 1.94	1.75 ± 2.13	1.67 ± 1.39	0.815	0.992	0.910	0.969
Interference with daily living ^8^	2.12 ± 1.77	2.49 ± 2.25	2.2 ± 2.02	3.36 ± 2.83	0.626	0.995	0.481	0.552
Physical activity score ^9^	58.59 ± 45.01	48.83 ± 26.6	46.18 ± 25.55	56.33 ± 30.53	0.692	0.855	0.999	0.763
Daily dietary intakes ^10^								
Dietary fiber, g	18.45 ± 5.71	18.5 ± 3.81	18.58 ± 4.17	18.29 ± 2.87	0.560	0.857	0.886	0.946
Calcium, mg	1036.6 ± 169	1036.7 ± 166.1	1051.6 ± 154.5	996.3 ± 201.7	0.948	0.985	0.854	0.462
Dairy, cup equivalent	1.59 ± 0.4	1.6 ± 0.38	1.59 ± 0.3	1.63 ± 0.56	0.969	0.999	0.978	0.902
Fruit, vegetables and legumes, cup equivalent	3.06 ± 1.22	3.15 ± 1.05	3.15 ± 1.05	3.16 ± 1.15	0.430	0.747	0.819	0.925
Fruits	1.06 ± 0.9	1.02 ± 0.49	1.12 ± 0.53	0.77 ± 0.27	0.344	0.363	0.961	0.182
Vegetables and legumes	1.83 ± 0.66	1.92 ± 0.81	1.83 ± 0.73	2.17 ± 1.01	0.990	0.962	0.886	0.715
Whole grains, ounce equivalent	0.73 ± 0.39	0.86 ± 0.39	0.95 ± 0.4	0.65 ± 0.27	0.204	0.210	0.995	0.250
Total added sugars, tsp	16.59 ± 4.13	17.49 ± 6.9	18.24 ± 7.57	15.46 ± 4.43	0.866	0.995	0.782	0.875
Added sugars from beverages	5.82 ± 2.23	7.64 ± 5.73	8.34 ± 6.47	5.74 ± 2.37	0.459	0.486	0.928	0.660

Abbreviations: MDASI, MD Anderson Symptom Inventory; tsp, teaspoon. ^1^ Includes patients with non-hormonal CT only, and patients with both hormonal and non-hormonal CT. ^2^ *p*-value based on Mann–Whitney U test for continuous variables and $\chi^{2}$ or Fisher’s exact test for categorical variables. ^3^ *p*-value based on Kruskal–Wallis test followed by Dwass–Steel–Critchlow–Fligner test for pairwise comparison for continuous variables, and $\chi^{2}$ or Fisher’s exact test for categorical variables. ^4^ Mean ± SD, all such values. ^5^ Center for Epidemiological Studies-Depression (CES-D): score ranging from 0 to 60, higher score indicating higher depressive symptomatology. ^6^ State-Trait Anxiety Inventory, state subscale (STAI-S): score ranging from 20 to 80, higher score indicating higher anxiety symptomatology. ^7^ Score ranging from 1 to 13, with higher scores indicating greater severity of symptoms. ^8^ Score ranging from 1 to 6, with higher scores indicating greater interference with living. ^9^ Godin–Shephard Leisure-Time Physical Activity Questionnaire. In our population, scores range from 3 to 170, with a higher score indicating higher physical activity levels. ^10^ Averaged intakes recalled over a 30-day period, assessed with the National Cancer Institute Dietary Screener Questionnaire (NCI-DSQ).

## Data Availability

The data that support the findings of this study are available from the corresponding authors upon request. Limitations apply to variables that may compromise participant privacy or consent.

## Supplementary Materials

The following supporting information can be downloaded at https://www.mdpi.com/article/10.3390/nu18010040/s1: Figure S1: Prevalence of immune-related adverse events and chronic toxicities reported by metastatic melanoma survivors; Figure S2: Association of gut microbiome with diet, physical activity and PROs in metastatic melanoma survivors.; Table S1: Treatment of metastatic melanoma survivors by chronic toxicities Table S2: Prevalence of patients with mild, moderate and severe symptoms in metastatic melanoma survivors; Table S3: *p*-values for correlations shown in Figure 2A; Table S4: *p*-values for correlations shown in Figure 2B.

## Abbreviations

The following abbreviations are used in this manuscript:

BMI	Body mass index
BH	Benjamini–Hochberg
CES-D	Center for Epidemiological Studies-Depression
CTs	Chronic toxicities
FDR	False discovery rate
ICB	Immune checkpoint blockade
irAEs	Immune-related adverse events
MDASI	MD Anderson Symptom Inventory
NCI-DSQ	National Cancer Institute Dietary Screener Questionnaire
PERMANOVA	Permutational multivariate analysis of variance
PROs	Patient-reported outcomes
QoL	Quality of life
SSBs	Sugar-sweetened beverages
STAI-S	State-Trait Anxiety Inventory, state subscale
