# Supplementary material for: Investigating Chronic Toxicity, Diet, Patient-Reported Outcomes and the Microbiome in Immunotherapy-Treated Metastatic Melanoma Survivors: A New Frontier
Source: Nutrients. 2025 Dec 22;18(1):40. doi: 10.3390/nu18010040 (PMC12787744; doi:10.3390/nu18010040)
Supplement: Supplementary file 1 [file nutrients-18-00040-s001.zip › nutrients-3991356-supplementary.pdf]

Supplemental Table S1. Treatment of metastatic melanoma survivors by chronic toxicities (N=48)

	Chronic toxicities		
	**No (n=19)**	**Hormonal only (n=21)**	**Other[1] (n=8)**
Treatment[2]			
Combination therapy	5 (26.3)	14 (66.7)	5 (62.5)
Monotherapy	13 (68.4)	7 (33.3)	2 (25)
Other	1 (5.3)	0.00	1 (12.5)

[1] *Includes uveitis, pneumonitis, pancreatitis, nephritis*

[2] *Combination therapy includes a combination of anti-PD-1 and anti-CTL-4 antibodies, Monotherapy includes either anti-PD-1 or anti-CTL-4 antibodies and other includes only targeted- and or chemotherapy.*

Supplemental Table S2. Prevalence of patients with mild, moderate and severe symptoms in metastatic melanoma survivors

**MDASI Item**	**Mild symptoms (rating ≤ 4)**	**Moderate symptoms (rating of 5-6)**	**Severe symptoms (rating ≥7)**
Pain, *n*%	40 (83.3)	2 (4.2)	6 (12.5)
Fatigue, *n*%	39 (81.2)	6 (12.5)	3 (6.3)
Nausea, *n*%	43 (95.6)	1 (2.2)	1 (2.2)
Disturbed sleep, *n*%	39 (86.7)	2 (4.4)	4 (8.9)
Distress/feeling upset, *n*%	42 (93.3)	2 (4.4)	1 (2.2)
Shortness of breath, *n*%	41 (91.1)	3 (6.7)	1 (2.2)
Difficulty remembering, *n*%	40 (88.9)	4 (8.9)	1 (2.2)
Lack of appetite, *n*%	42 (93.3)	1 (2.2)	2 (4.4)
Drowsiness, *n*%	41 (91.1)	3 (6.7)	1 (2.2)
Dry mouth, *n*%	46 (95.8)	1 (2.1)	1 (2.1)
Sadness, *n*%	43 (95.6)	1 (2.2)	1 (2.2)
Vomiting, *n*%	44 (97.8)	0 (0)	1 (2.2)
Numbness/tingling, *n*%	38 (84.4)	4 (8.9)	3 (6.7)

Abbreviation: MDASI, MD Anderson Symptom Inventory

Supplemental Table S3: P-values for correlations shown in Figure 2.A.

		Fiber	Calcium	Dairy	Fruit, vegetables and legumes	Fruits	Vegetables and legumes	Whole grains	Total added sugars	Physical activity	BMI
**Absence of CT**	Depressive symptoms score	0.112	0.216	0.184	0.410	0.017	0.628	0.093	0.418	**0.072**	0.413
	Anxiety symptoms score	0.277	0.566	0.416	0.663	0.352	0.744	0.413	0.331	0.078	0.414
	Severity of symptoms	0.534	0.367	0.088	0.724	0.019	0.355	0.716	0.158	0.909	0.316
	Interference with daily living	0.122	0.835	0.968	0.466	0.060	0.768	0.253	0.799	0.908	0.166
**Presence of CT**	Depressive symptoms score	0.572	0.841	0.514	0.697	0.195	0.766	0.550	0.675	0.532	0.955
	Anxiety symptoms score	0.435	0.473	0.598	0.169	0.485	0.046	0.279	0.788	0.640	0.683
	Severity of symptoms	0.989	0.798	0.726	0.606	0.497	0.756	0.123	0.036	0.007	0.447
	Interference with daily living	0.532	0.572	0.992	0.317	0.501	0.488	0.216	0.092	0.056	0.209
**Complete sample**	Depressive symptoms score	0.560	0.388	0.105	0.881	0.939	0.393	0.665	0.801	0.088	0.663
	Anxiety symptoms score	0.193	0.245	0.259	0.219	0.368	**0.038**	0.169	0.798	0.358	0.932
	Severity of symptoms	0.883	0.529	0.148	0.799	0.143	0.690	0.455	**0.003**	**0.046**	0.148
	Interference with daily living	0.183	0.510	0.883	0.425	0.210	0.435	0.108	0.116	0.175	0.079

Supplemental Table S4: P-values for correlations shown in Figure 2.B.

Symptoms[1]	Absence of CT (n=19)		Presence of CT (n=29)		Complete sample (n=48)	
	**Total added sugars**	**Added sugars from SSBs**	**Total added sugars**	**Added sugars from SSBs**	**Total added sugars**	**Added sugars from SSBs**
Pain	0.4702	0.8534	0.0280	0.0023	0.0158	0.0068
Fatigue	0.1469	0.5416	0.0919	0.0159	0.0188	0.0164
Nausea	0.3476	0.7695	0.1810	0.0227	0.0829	0.026
Disturbed sleep	0.2237	0.3350	0.1459	0.0399	0.0498	0.0345
Distress/feeling upset	0.7710	0.4074	0.0622	0.0372	0.1351	0.1905
Shortness of breath	0.1801	0.5652	0.0087	0.0184	0.0029	0.0138
Difficulty remembering	0.4044	0.7565	0.0809	0.0866	0.0536	0.1052
Lack of appetite	0.8387	0.5645	0.0072	0.0033	0.0324	0.04
Drowsiness	0.0018	0.0348	0.0708	0.0048	0.0036	0.0015
Dry mouth	0.1429	0.0410	0.1682	0.0335	0.0349	0.011
Sadness	0.4814	1.0000	0.1347	0.2575	0.0751	0.3129
Vomiting	0.3993	0.3230	0.0042	0.0002	0.0038	0.0013
Numbness/tingling	0.2488	0.9315	0.0778	0.0477	0.0248	0.1293
General activity	0.2129	0.4698	0.1644	0.0120	0.047	0.0167
Mood	0.5589	0.2495	0.2043	0.0322	0.1611	0.0132
Working (incl. housework)	0.9582	0.1596	0.1553	0.0131	0.1985	0.0056
Relations with other people	0.6841	0.3913	0.1807	0.0456	0.3615	0.0334
Walking	0.4484	0.5987	0.0326	0.0062	0.0186	0.0081
Enjoyment of life	0.8907	0.4189	0.1010	0.0368	0.1667	0.0251

[1] **As measured by the MD Anderson Symptom Inventory**

Supplementary Figure S1. Prevalence of immune-related adverse events and chronic toxicities reported by metastatic melanoma survivors.

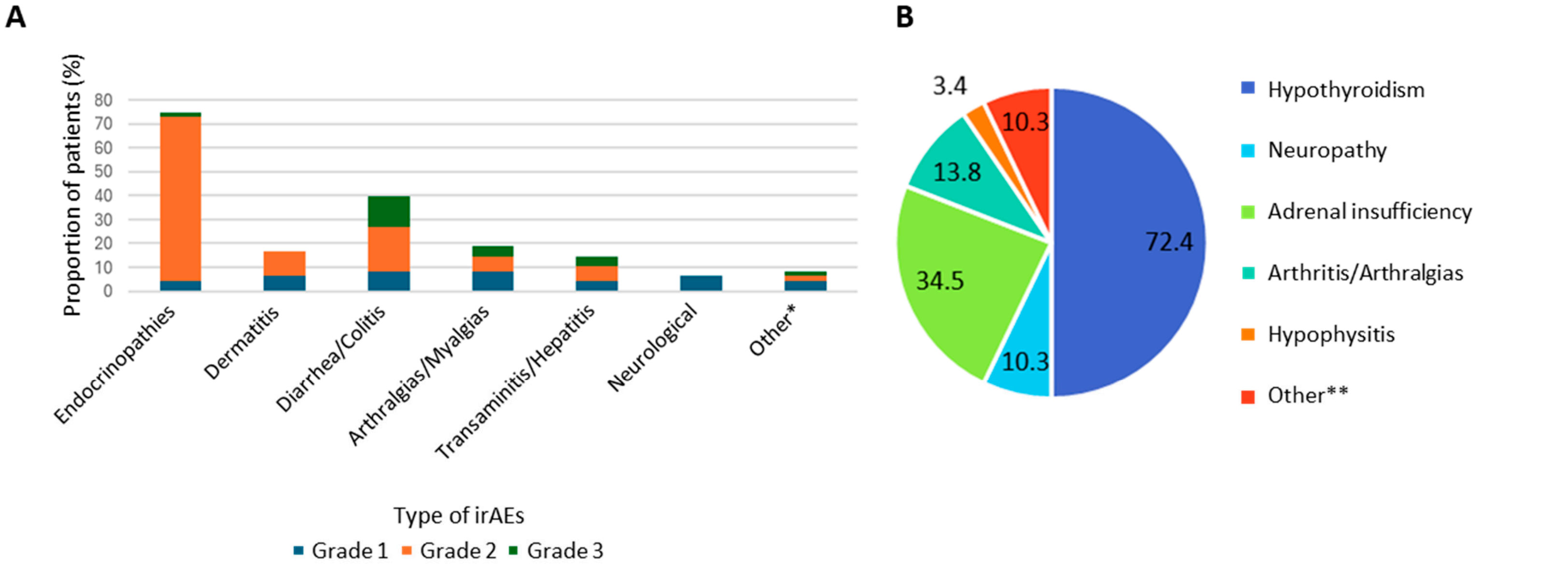

A. Immune-related adverse events (irAEs) during treatment period, prior to the current study (%), total n=48. **B.** Prevalence of chronic toxicity (CT) persisting after completion of therapy (%), total n=29.

* Other category includes 2 cases of uveitis, 1 pneumonitis, 1 pancreatitis, 1 nephritis.

** Other category includes 1 case of ongoing vitrisis, 1 lump vitiligo and 1 low levels of vitamin D and calcium.

Supplementary Figure S2. Association of gut microbiome with diet, physical activity and PROs in metastatic melanoma survivors.

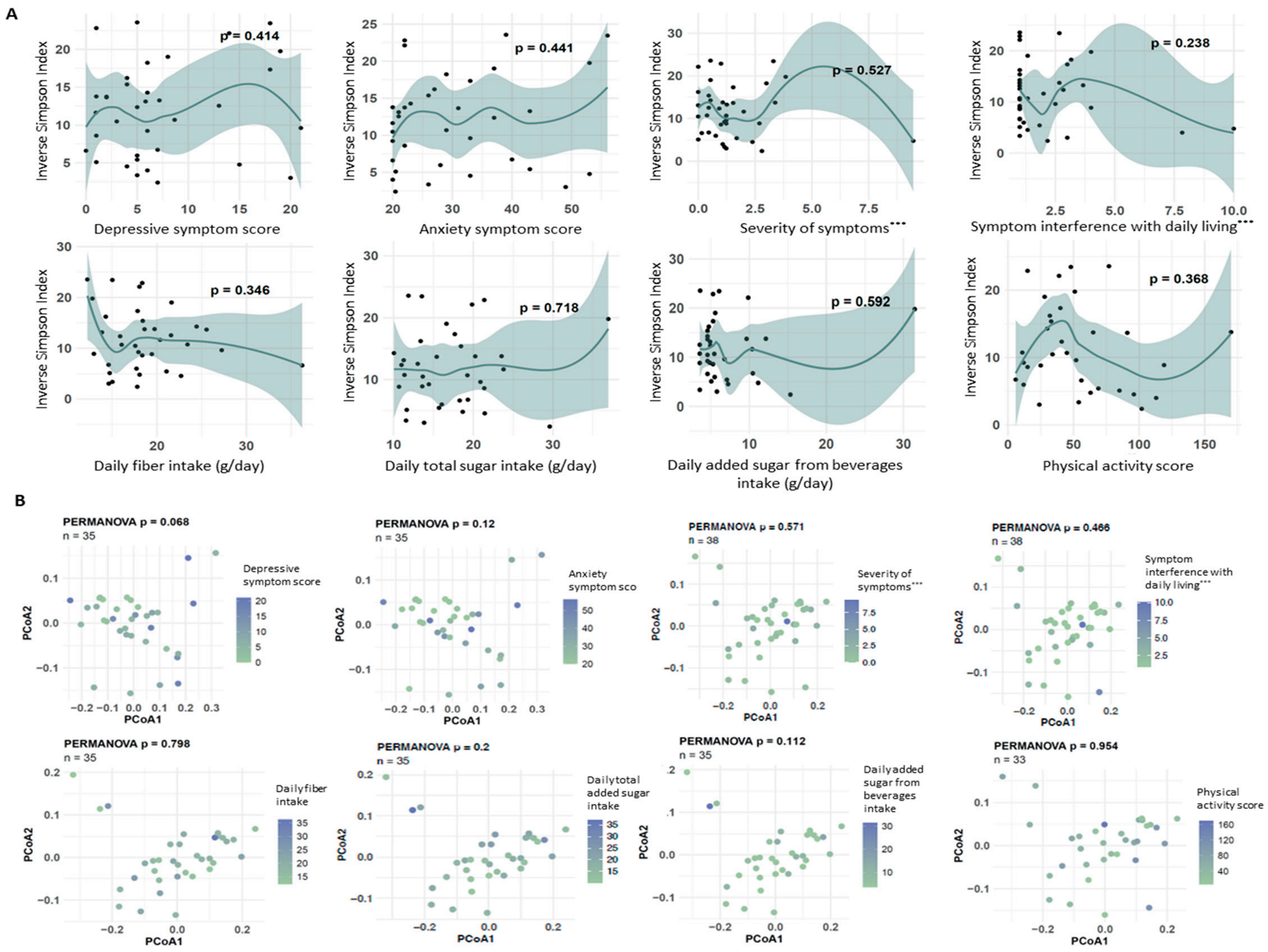

A, Plot of alpha diversity alpha diversity (Inverse Simpson index) across PRO and lifestyle variables; **B,** Principal Coordinates Analysis (PCoA) plots of beta-diversity in microbial composition across PROs and lifestyle variables.
*** as measured by the MD Anderson Symptom Inventory.
